# Vaginal Microbiota Changes in Patients With Premature Ovarian Insufficiency and Its Correlation With Ovarian Function

**DOI:** 10.3389/fendo.2022.824282

**Published:** 2022-02-22

**Authors:** Jingyi Wen, Yanzhi Feng, Wei Yan, Suzhen Yuan, Jinjin Zhang, Aiyue Luo, Shixuan Wang

**Affiliations:** ^1^ Department of Obstetrics and Gynecology, Tongji Hospital, Tongji Medical College, Huazhong University of Science and Technology, Wuhan, China; ^2^ National Clinical Research Center for Obstetrical and Gynecological Diseases, Tongji Hospital, Tongji Medical College, Huazhong University of Science and Technology, Wuhan, China; ^3^ Key Laboratory of Cancer Invasion and Metastasis, Ministry of Education, Wuhan, China

**Keywords:** premature ovarian insufficiency (POI), premature ovarian failure (POF), vaginal microbiota, ovarian reserve, ovarian endocrine function, perimenopausal syndromes

## Abstract

**Objectives:**

To reveal the characteristics of vaginal microbiota in premature ovarian insufficiency (POI) patients and their relationship with ovarian function.

**Materials and Methods:**

In this case-control study, the vaginal bacterial composition of 30 POI patients and 26 healthy women of comparable age was assessed by 16S rRNA gene sequencing targeting the V3-V4 hypervariable regions. The metabolic functions of vaginal microflora were preliminarily predicted through the PICRUSt2 analysis. Redundancy analysis and Spearman’s correlation analyzed the relationships between vaginal microbiota and ovarian function indicators.

**Results:**

*Actinobacteria*, *Atopobium*, and *Gardnerella* were significantly increased in POI patients. Their increments were significantly negatively correlated with anti-müllerian hormone (AMH) and inhibin B, and positively correlated with follicle-stimulating hormone (FSH) and luteinizing hormone (LH). While *Bifidobacterium* was significantly decreased in POI patients. Its relative abundance was significantly positively correlated with AMH and negatively correlated with FSH and LH. Then, POI patients included in this study were divided into POI (25 < FSH ≤ 40) (*n* = 9) and premature ovarian failure (POF) (FSH > 40) (*n* = 21) subgroups according to serum FSH levels. Compared with the controls, *Firmicutes* and *Lactobacillus* were significantly decreased only in POF (FSH > 40) patients, while no difference was observed in POI (25 < FSH ≤ 40) patients. *Lactobacillus* was negatively correlated with FSH. *Firmicutes* was significantly reduced and *Actinobacteria* was significantly increased in POF (FSH > 40) patients compared with POI (25 < FSH ≤ 40) patients. The key bacterial taxa *Gardnerella* and *Atopobium* showed potency in predicting POI.

**Conclusions:**

Here we demonstrated significant changes in the vaginal microbiota of POI patients, and these changes were significantly correlated with reduced ovarian reserve, endocrine disruption, and symptoms of perimenopausal syndrome. Differences in vaginal microbiota between POI (25 < FSH ≤ 40) and POF (FSH > 40) patients were also identified. These findings may provide new evidence for the relationship between vaginal microbiota and ovarian function.

## Introduction

Premature ovarian insufficiency (POI) is a medical condition in which ovarian follicles are depleted and cease to function normally in women under 40 years of age (1). The clinical manifestation differs between individuals, mainly including menstrual disturbance, subfertility or infertility, and symptoms of perimenopausal syndrome (2). Moreover, long-term consequences of premature loss of ovarian function have a significant negative impact on psychological wellbeing and quality of life; it also increases the risk of osteoporosis, and cardiovascular and neurological disorders (1, 3–6). Although multiple causes have been reported, including genetic (7–10), autoimmune (11, 12), iatrogenic (13–15), and environmental pollutants (16, 17), et al, the exact etiology of POI remains unclear. Over half of patients are diagnosed with idiopathic POI. Therefore, considering the serious adverse effects of POI on female reproductive and general health, it is of great importance to study POI.

Researchers have begun to pay attention to the impact of gut and vaginal microbiomes on female reproductive endocrine function (18). Studies that evaluated the effect of gut microbiota on ovarian function are limited and mainly focus on polycystic ovarian syndrome (PCOS) and menopause (19, 20). Recently, one study revealed different abundances of *Eggerthella* in the gut, serum levels of metabolites, and transforming growth factor-β1 (TGF-β1) in POI patients, and these alterations could be reversed by hormone replacement therapy (HRT) (21). As an important part of the female reproductive tract, there have been some new findings on the relationship between vaginal microbiota and ovarian function. Early studies established the paradigm that, following menopause, vaginal *Lactobacillus* decreases, and overall bacterial levels and diversity are altered (22). Similar results have been observed in women with POF (23, 24). POI, POF, early menopause, and natural menopause reflect different stages of ovarian aging. With FSH > 40 IU/L as the diagnostic threshold, POF can only represent the terminal stage of ovarian aging. With a lower diagnostic threshold of FSH > 25 IU/L, POI can achieve the earlier diagnosis and timely treatment of ovarian function failure compared with POF (2, 25). However, whether there had already been a change in the vaginal microbiota during the POI stage, and the relationship between vaginal microbiota alteration and ovarian function, still need to be confirmed.

In this study, we compared the diversity, composition, and function of vaginal microbiota in POI patients with healthy women of comparable age. Our results revealed the correlations between vaginal microbiota and indicators of ovarian reserve and ovarian function. The identified bacteria and their metabolic function differences may provide important insights into further research regarding the microbiome in the pathophysiology, diagnosis, and treatment of POI.

## Subjects and Methods

### Study Population

This study set out to describe the vaginal microbiota of POI patients. POI is defined as oligo/amenorrhea for at least four months before age 40 years, and an elevated FSH level > 25 IU/L on two occasions > four weeks apart (2). Women younger than 40 years with regular menstruation and normal ovarian function were included as the control group. Exclusion criteria were as follows: (1) women who are pregnant or lactating, (2) women who had taken exogenous sex hormones in the previous three months, (3) women who had received antibiotics, probiotics, prebiotics, intravaginal drugs, etc. in the previous month, (4) women who are suffering from bacterial vaginosis, pelvic inflammatory disease, and other reproductive system infection diseases, (5) women who are suffering from serious diseases and who required long-term medical treatment, (6) women who are participating in other clinical trials. This study was approved by the Ethical Committee of Tongji Medical College, Huazhong University of Science and Technology. All participants agreed to join the study and signed their informed consent.

### Sample and Data Collection

A total of 30 eligible POI patients and 26 controls were included in the Menopause & Ovarian Aging Specialty Clinic of Tongji Hospital. Participants’ basic clinical data were obtained from questionnaires and electronic medical records by experienced interviewers. The information included demographic characteristics, lifestyles, reproductive history, menstrual history, Kupperman index (KMI) scale, hospital anxiety and depression (HAD) scale. Only three POI patients had received HRT in the past, and the others were all newly diagnosed. Two of the POI patients still had menstrual cycles, while the others had amenorrhea or menopause.

Blood samples were collected from day 2 to 5 of the latest menstrual cycle for women who still had menstrual cycles. For women with amenorrhea or menopause, blood samples were also collected for serum hormones detection. Vaginal swab samples were collected for each participant by experienced gynecologists (avoiding menstruation for women who still had menstrual cycles). All samples were obtained in compliance with clinical ethics regulations, and the principle of sterility was strictly observed during the sampling process. All samples were stored at -80°C until assayed. Specimens from all participants were processed similarly in terms of sample collection, storage, DNA extraction, library preparation, and sequencing.

### Serum Hormones Detection

Serum levels of FSH (mIU/mL), LH (mIU/mL), prolactin (PRL, ng/ml), progesterone (PRG, ng/ml), testosterone (T, ng/ml), and estradiol (E2, pg/ml) were measured using a chemiluminescence-based immunometric assay on an ADVIA Centaur immunoassay system (Siemens Healthcare Diagnostics Inc., Tarrytown, NY, USA). The manipulation was performed following the manufacturer’s instructions. Serum levels of AMH (ng/ml) and inhibin B (pg/ml) were measured using AMH and inhibin B ELISA kits (Beckman Coulter Inc., Sacramento, CA, USA). Protocols were described in previous studies (26, 27).

### DNA Extraction and 16S rRNA Gene Sequencing

Genomic DNA was extracted from archived vaginal swab specimens using an E.Z.N.A. ^®^Stool DNA Kit (D4015, Omega Inc., Norcross, GA, USA) according to the manufacturer’s instructions. The V3-V4 hypervariable regions of the bacterial small-subunit (16S) rRNA gene were amplified with slightly modified versions of primers 341F (5’-CCTACGGGNGGCWGCAG-3’) and 805R (5’-GACTACHVGGGTATCTAATCC-3’). A library was constructed and amplified products sequenced on an Illumina NovaSeq PE250 platform (LC-Bio Technology Co., Ltd, Hang Zhou, China). Paired-end reads were assigned to samples based on their unique barcode and truncated by cutting off the barcode and primer sequence. Paired-end reads were merged using FLASH (v1.2.8). Quality filtering on the raw tags was performed under specific filtering conditions to obtain high-quality clean tags according to fqtrim (v0.94). Chimeric sequences were filtered using VSEARCH software (v2.3.4). After dereplication using DADA2, we obtained a feature table and feature sequence. Alpha diversity and beta diversity were calculated by normalized to the same sequences randomly. Subsequently, according to SILVA (release 132) classifier, feature abundance was normalized using the relative abundance of each sample. Blast was used for sequence alignment, and the feature sequences were annotated with SILVA (release 132) and NT-16S database for each representative sequence. Cluster analysis and LEfSe analysis were conducted to compare differences in the composition of the vaginal microbiota. Functional pathways of vaginal microbiota in the two groups were predicted by PICRUSt2 analysis. Redundancy analysis (RDA) was used to evaluate the important relationship between bacterial taxa and environmental factors. Bioinformatic analysis was performed using the OmicStudio tools at http://www.omicstudio.cn/tool.

### Statistical Analysis

The Shapiro-Wilk test was conducted to test the distribution types of continuous variables. Continuous variables conforming to normal distribution were presented as mean ± SD (standard deviation) and compared by group using the Student’s *t*-test. Meanwhile, variables with skew distribution were expressed as median (interquartile, IQR) or median (range) and compared using the nonparametric Wilcoxon rank-sum test. Categorical variables were expressed as frequencies (percentages, %) and compared using the chi-square test. KMI scores ranging from 0–6, 7–15, and > 16 were used to rate the degree of severity as none, mild, and moderate to severe, respectively (28). HAD scores ranging from 0–7, and > 7 represented normal, and anxiety/depression, respectively (29).

Spearman’s correlation was used to analyze the correlations between the relative abundance of vaginal microbiota and clinical indicators related to ovarian reserve, ovarian endocrine function, and symptoms of perimenopausal syndrome. Meanwhile, for each important phylum or genus with significant differences, we constructed the receiver operating characteristic (ROC) curve and computed the area under the curve (AUC) value. A two-tailed *P*<0.05 was considered statistically significant. All data were analyzed using IBM SPSS Statistics 20.0 (SPSS Inc., Chicago, IL, USA).

## Results

### Basic Clinical Characteristics of the Study Population

The age at menarche in the POI group was significantly lower than that of the controls (*P* = 0.016), and the proportion of passive smoking exposure history was significantly higher than that in the control group (*P* = 0.045) (**Table S1**). Whereas no significant difference was found in age and BMI, as did other demographic characteristics, lifestyles, reproductive histories, and HAD scores (*P* > 0.05). Moreover, FSH and LH were significantly higher in the POI group (*P* < 0.001). Serum levels of T, E2, AMH, and inhibin B were lower among POI patients than the controls (*P* < 0.001, *P* < 0.001, *P* < 0.001, *P* < 0.001, respectively). Levels of PRL and PRG were not significantly different between the two groups (*P* > 0.05). POI patients had significantly higher KMI scores than the controls (*P* = 0.001), indicating more serve symptoms of perimenopausal syndrome in the POI group (*P* = 0.003). These differences were basically in line with the clinical phenotype and pathophysiological characteristics of POI.

### Alterations in the Diversity and Composition of Vaginal Microbiota in POI Patients

No significant difference was found in the alpha diversity of vaginal microbiota between POI patients and the controls (**Figures S1A–E**); whereas, beta diversity was significantly different between the two groups according to Anosim (analysis of similarities, *R* = 0.0634, *P* = 0.027) and was displayed in PCA (principal component analysis) plots (**Figure S1F**).

**Figure 1:**
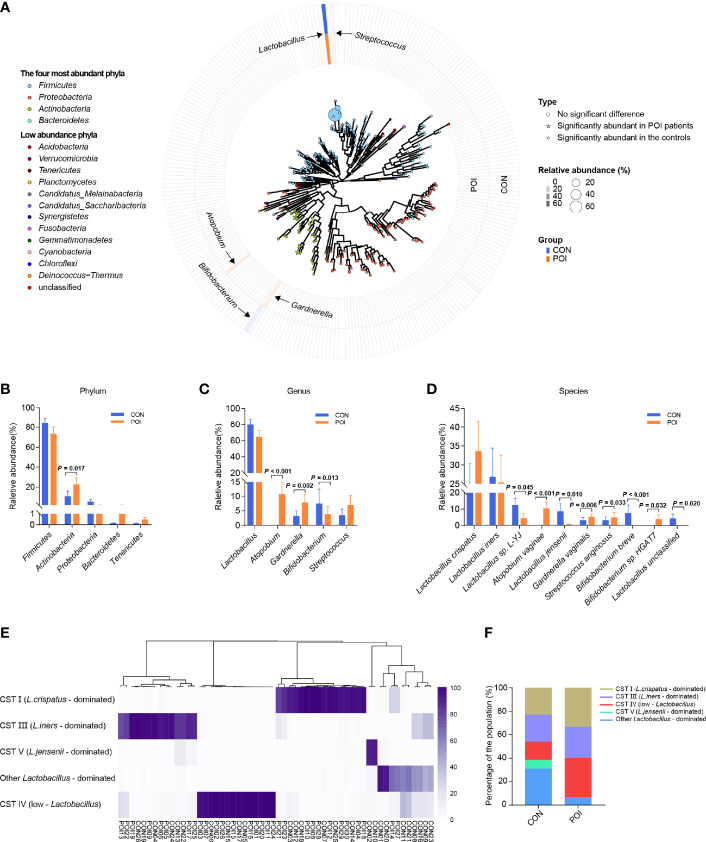
Comparison of vaginal microbiota composition of women with POI and healthy controls. **(A)** A map of vaginal microbiota composition in all samples. The vaginal microbiome is dominated by four phyla: *Firmicutes*, *Actinobacteria*, *Proteobacteria*, and *Bacteroidetes*. In the center is a phylogenetic tree of microbes abundant in the vaginal microbiota. Bacteria significantly abundant in POI patients are indicated by stars, bacteria abundant in the controls are indicated by triangles, and those that have no significant difference are indicated by circles. A larger star/triangle/circle at the end indicates a higher relative abundance of the corresponding genus. The outer rings correspond to the relative abundance of each genus in each group and are color-coded by group, with darker colors indicating higher relative abundance. **(B–D)** Differences in relative abundance at phylum, genus, and species levels. **(E)** Cluster analysis of dominant vaginal bacterial taxa found in 56 participants. **(F)**. Percentage of each cluster in the two groups. CST, community state type. *P* < 0.05 was considered statistically significant.

The dominant vaginal bacteria in both POI patients and the controls were *Firmicutes* at the phylum level and *Lactobacillus* at the genus level (**Figure S2** and **Tables S2**, **S3**). The four most abundant phyla in all samples were *Firmicutes*, *Actinobacteria*, *Proteobacteria*, and *Bacteroidetes* (**Figure 1A**). The top five bacterial genera in relative abundance were *Lactobacillus*, *Atopobium*, *Gardnerella*, *Bifidobacterium*, and *Streptococcus*, respectively belonging to *Firmicutes* and *Actinobacteria*. Moreover, at the phylum level, POI patients had a significantly greater abundance of *Actinobacteria* (22.58% vs 10.65%, *P* = 0.017), while the abundance of *Firmicutes* was lower, it was not statistically significant (73.21% vs 83.99%, *P* = 0.172) (**Figure 1B**). At the genus level, the relative abundance of *Atopobium* and *Gardnerella* were significantly increased in POI patients (10.67% vs 0.01%, *P* < 0.001; 7.81% vs 3.13%, *P* = 0.002), while the relative abundance of *Bifidobacterium* was significantly decreased (3.81% vs 7.44%, *P* = 0.013). The relative abundance of *Lactobacillus* decreased in POI patients but showed no significant difference between the two groups (64.12% vs 79.54%, *P* = 0.172) (**Figure 1C**). However, a heatmap visualizing Spearman’s Rho correlation coefficients of the top 20 abundant genera revealed that *Lactobacillus* was negatively correlated with all other genera (**Figure S3**). *Atopobium* and *Gardnerella*, which were significantly increased in POI patients, were positively correlated to each other, and they were both negatively correlated to *Bifidobacterium*, which was significantly enriched in the control group.

At the species level, the relative abundance of *Lactobacillus* sp. L-YJ, *Lactobacillus jensenii*, *Bifidobacterium breve*, and *Lactobacillus unclassified* were significantly decreased in POI patients (*P* = 0.045, *P* = 0.010, *P* < 0.001, *P* = 0.020, respectively), while the relative abundance of *Atopobium vaginae*, *Gardnerella vaginalis*, *Streptococcus anginosus*, and *Bifidobacterium* sp. HGAT7 were significantly increased (*P* < 0.001, *P* = 0.006, *P* = 0.033, *P* = 0.032, respectively) (**Figure 1D**, **Table S4**). Hierarchical clustering of bacterial community composition data showed that the 56 vaginal samples could be assigned to five distinct clusters based on differences in the composition of relative abundance of bacterial taxa (**Figure 1E**). Community state type (CST) I (*n* = 16), CST III (*n* = 14), and CST V (n = 2) were dominated by *Lactobacillus crispatus*, *Lactobacillus iners*, and *Lactobacillus jensenii*, respectively, which were consistent with the results of Ravel J (30). Another group was dominated by other *Lactobacillus* species (n = 10), including *Lactobacillus* sp. L-YJ and uncultured *Lactobacillus* sp. CST IV (n = 14) was typified by higher proportions of strictly anaerobic bacteria, including *Atopobium*, *Streptococcus*, and *Gardnerella* (30). In this study, *Lactobacillus* was still the dominant strain in most of the population (66.67% in POI patients, 84.62% in controls), while the proportion of CST IV in the POI group was higher than that in the control group (33.33% vs 15.38%), but the difference was not significant (*P* = 0.122) (**Figure 1F**).

### Comparison of Vaginal Microbiota Composition Between POI (25 < FSH ≤ 40) and POF (FSH > 40) Subgroups

**Figure 2:**
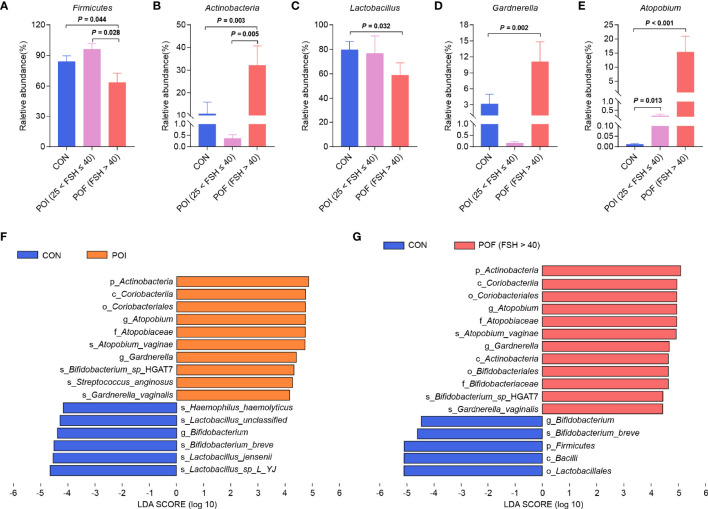
Comparison of vaginal microbiota composition between POI (25 < FSH ≤ 40) and POF (FSH > 40) subgroups. **(A–E)** Differences in relative abundance of *Firmicutes*, *Actinobacteria*, *Lactobacillus*, *Gardnerella*, and *Atopobium*. **(F)**. Histogram of the LDA scores for differentially abundant bacterial taxa between the control and the POI groups (LDA > 4). **(G)**. Histogram of the LDA scores for differentially abundant bacterial taxa between the control group and the POF (FSH > 40) subgroup (LDA > 4). LDA, linear discriminant analysis. *P* < 0.05 was considered statistically significant.

Instead of the previous diagnostic threshold of FSH > 40 IU/L in the past, the 2016 European Society of Human Reproduction and Embryology (ESHRE) guidelines lowered it to 25 IU/L to achieve the goal of early detection and timely treatment (2). POI patients included in this study were divided into POI (25 < FSH ≤ 40) and POF (FSH > 40) subgroups according to serum FSH levels (2, 25) (**Figures 2A–E**). When compared with the control group, the relative abundance of *Firmicutes*, and *Lactobacillus* were significantly reduced only in POF (FSH > 40) patients (*n* = 21) (63.41% vs 83.99%, *P* = 0.044; 58.77% vs 79.54%, *P* = 0.032). The relative abundance of *Actinobacteria*, *Atopobium*, and *Gardnerella* were also significantly increased mainly in POF (FSH > 40) patients (32.10% vs 10.65%, *P* = 0.003; 15.58% vs 0.01%, *P* < 0.001; 11.08% vs 3.14%, *P* = 0.002). Interestingly, when compared with POI (25 < FSH ≤ 40) subgroup (*n* = 9), POF (FSH > 40) patients had significantly lower relative abundance of *Firmicutes* and higher relative abundance of *Actinobacteria* (63.41% vs 96.09%, *P* = 0.028; 32.10% vs 0.36%, *P* = 0.005). The relative abundance of *Lactobacillus* was lower in POF (FSH > 40) patients, and the relative abundances of *Atopobium*, and *Gardnerella* were higher, but the differences were not significant (58.77% vs 76.53%, *P* = 0.099; 15.28% vs 0.17%, *P* = 0.389; 11.08% vs 0.15%, *P* = 0.141). Taken together, our results suggested that not only did the composition of vaginal microbiota change significantly in POI patients, but POI (25 < FSH ≤ 40) and POF (FSH > 40) patients also had some differences in vaginal microbiota composition.

### Identification of Key Bacterial Taxa Associated With POI and Prediction of Their Metabolic Functions

LEfSe analysis was performed to identify the specific and key bacterial taxa associated with POI (**Figure 2F**). At the phylum level, *Actinobacteria* was significantly enriched in the POI patients. At the genus level, *Atopobium* and *Gardnerella* were dramatically enriched in the POI group. The results for control and POF (FSH > 40) groups were similar to the above results, except for the significant enrichment of *Firmicutes* in the control group (**Figure 2G**). Collectively, these might be potential microbiological markers for discriminating patients with POI.

**Figure 3:**
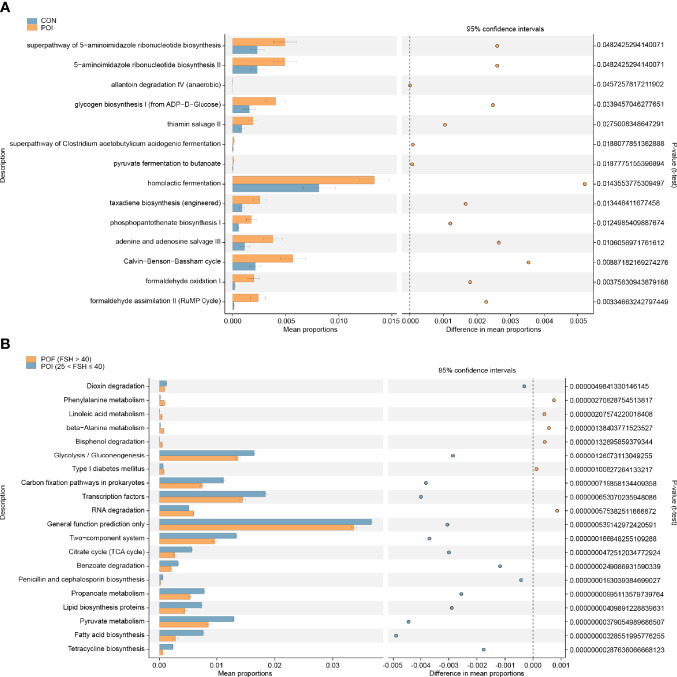
Analyze and predict the metabolic functions of vaginal microbiota by the PICRUSt2 method. **(A)** Significantly different pathways between the POI and the control groups. **(B)** Top 20 significantly different pathways between the POI (25 < FSH ≤ 40) and POF (FSH > 40) subgroups. PICRUSt, phylogenetic investigation of communities by reconstruction of unobserved states. *P* < 0.05 was considered statistically significant.

Thereafter, we preliminarily predicted the metabolic functions of vaginal microflora using the PICRUSt2 analysis. STAMP analysis identified 14 significantly different pathways between the POI and the control groups (**Figure 3A**). Homolactic fermentation was the most enriched pathway in POI patients. Significant changes were also observed when the POI (25 < FSH ≤ 40), and POF (FSH > 40) subgroups were compared with the control group, respectively (**Figure S4**). Moreover, there were significant differences between the POI (25 < FSH ≤ 40) subgroup and the POF (FSH > 40) subgroup (**Figure 3B**). Among the top 20 significant pathways, phenylalanine metabolism, linoleic acid metabolism, beta-alanine metabolism, bisphenol degradation, type I diabetes mellitus, and RNA degradation were significantly enriched in POF (FSH > 40) patients.

### Correlation Analysis of Vaginal Microbiota With Ovarian Function

Because age at menarche and passive smoking exposure history were significantly different between groups, we also analyzed their association with vaginal microbiota (**Table S5**). Passive smoking was significantly negatively correlated with observed-OTUs and Chao1 index (*r* = -0.34, *P* = 0.010; *r* = -0.35, *P* = 0.009), and positively correlated with the relative abundance of *Bifidobacterium* (*r* = 0.28, *P* = 0.038). No correlation was found between age at menarche and vaginal microbiota.

**Figure 4:**
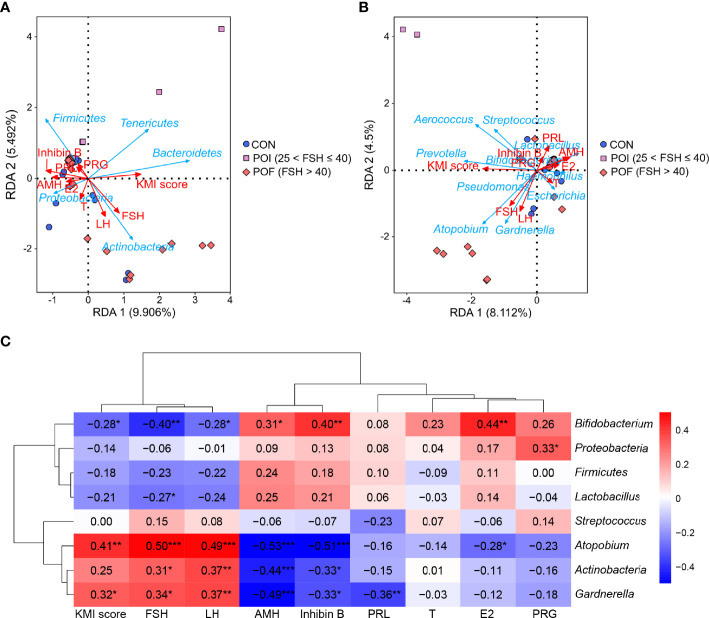
Correlation analysis of vaginal microbiota with ovarian reserve, ovarian function, and KMI score. **(A, B)** RDA revealed the important relationships between the top five phyla, the top ten genera, and clinical indicators related to ovarian reserve, ovarian function, and KMI score. **(C)** Spearman’s correlations between the relative abundance of vaginal microbiota and ovarian reserve, ovarian function, and KMI score. KMI, Kupperman index; FSH, follicle-stimulating hormone; LH, luteinizing hormone; AMH, anti-müllerian hormone; PRL, prolactin; T, testosterone; PRG, progesterone; E2, estradiol; RDA, redundancy analysis. *P* < 0.05 was considered statistically significant. ^*^ *P* < 0.05, ^**^ *P* < 0.01, ^***^ *P* < 0.001.

The important relationships between the top five phyla, the top ten genera, and clinical indicators related to ovarian reserve (AMH, inhibin B), ovarian function (FSH, LH, PRL, PRG, T, and E2), and symptoms of perimenopausal syndrome (KMI score) were studied with redundancy analysis (**Figures 4A, B**). Serum FSH and LH levels, which were usually increased dramatically in POI patients, were closely associated with vaginal microbiota, as was the KMI score. They were both positively correlated to *Actinobacteria*, *Gardnerella*, and *Atopobium*, and negatively correlated to *Firmicutes*, *Lactobacillus*, and *Bifidobacterium*. While AMH and inhibin B, which were usually decreased in POI patients, were both negatively correlated with *Actinobacteria*, *Gardnerella*, and *Atopobium*, and positively correlated with *Firmicutes*, *Lactobacillus*, and *Bifidobacterium*.

Then, Spearman’s correlation coefficients between vaginal microbiological characteristics (alpha diversity indices, the relative abundance of the top three phyla and the top five genera) and ovarian function were further calculated. No significant correlation was found between alpha diversity and these clinical indicators (*P* > 0.05) (**Figure S5**), as neither were the relative abundance of *Firmucutes* and *Streptococcus* (**Figure 4C**). The relative abundance of *Actinobacteria*, *Gardnerella*, and *Atopobium* were all significantly negatively correlated with serum AMH (*r* = -0.44, *P* < 0.001; *r* = -0.49, *P* < 0.001; *r* = -0.53, *P* < 0.001) and inhibin B (*r* = -0.33, *P* = 0.012; *r* = -0.33, *P* = 0.012; *r* = -0.51, *P* < 0.001) levels, while significantly positive correlations were found in *Bifidobacterium* (*r* = 0.31, *P* = 0.019; *r* = 0.40, *P* = 0.002). The relative abundances of *Actinobacteria*, *Gardnerella*, and *Atopobium* were all significantly positively correlated with serum FSH (*r* = 0.31, *P* = 0.020; *r* = 0.34, *P* = 0.010; *r* = 0.50, *P* < 0.001) and LH (*r* = 0.37, *P* = 0.005; *r* = 0.37, *P* = 0.005; *r* = 0.49, *P* < 0.001) levels, while *Bifidobacterium* was significantly negatively correlated with them (*r* = -0.40, *P* = 0.002; *r* = -0.28, *P* = 0.040). The relative abundance of *Lactobacillus* was also negatively correlated with serum FSH levels (*r* = -0.27, *P* = 0.046). E2 is one of the most important steroid hormones secreted by the ovaries, and its level is significantly reduced in POI patients. The relative abundance of *Atopobium* was significantly negatively correlated with serum E2 levels (*r* = -0.28, *P* = 0.037), while *Bifidobacterium* was significantly positively correlated with serum E2 levels (*r* = 0.44, *P* = 0.001). The KMI score reflects the severity of the symptoms of perimenopausal syndrome. We found that the relative abundance of *Gardnerella* and *Atopobium* were both significantly positively correlated with the KMI score (*r* = 0.32, *P* = 0.016; *r* = 0.41, *P* = 0.002). Altogether, the increase of *Gardnerella* and *Atopobium*, and the decrease of *Lactobacillus*, and *Bifidobacterium* in vaginal microbiota were significantly correlated with reduced ovarian reserve, endocrine disruption, and symptoms of perimenopausal syndrome in POI patients.

### The Potential of Key Bacterial Taxa in the Vagina to Predict POI

**Figure 5:**
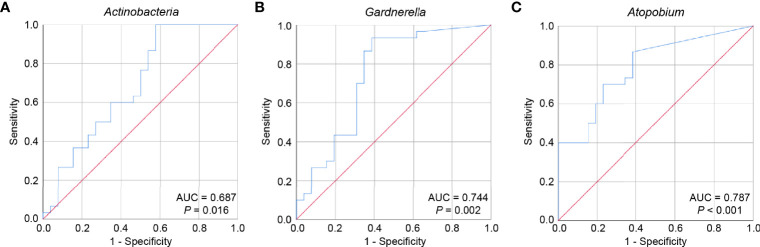
The potential of key bacterial taxa to predict POI. ROC curves were constructed and AUC values were calculated to assess the potential of predicting POI for *Actinobacteria* **(A)**, *Gardnerella* **(B)**, and *Atopobium* **(C)**. ROC, receiver operating characteristic; AUC, area under the curve.

To assess the potential of key bacterial taxa in predicting POI, ROC curves were constructed and AUC values were calculated for *Actinobacteria*, *Gardnerella*, and *Atopobium*. Given that an AUC greater than 0.7 is considered acceptable (31), the accuracies of *Gardnerella* and *Atopobium* in predicting POI were considered acceptable (AUC = 0.744, *P* = 0.002; AUC = 0.787, *P* < 0.001), while the accuracy of *Actinobacteria* was relatively low (AUC = 0.687, *P* = 0.016) (**Figure 5**). The accuracy of *Gardnerella* (AUC = 0.762, *P* = 0.002) and *Atopobium* (AUC = 0.795, *P* = 0.001) in predicting POF (FSH > 40) were higher than POI, while that of *Actinobacteria* (AUC = 0.658, *P* = 0.031) were lower than POI (**Figure S6**). Only *Atopobium* had a predictive value for POI (25 < FSH ≤ 40) (AUC = 0.769, *P* = 0.017) (**Figure S7**). Collectively, our results indicated that the key bacterial taxa showed certain potential in predicting POI and POF (FSH > 40), and more other relevant indicators combined may improve early detection efficiency.

## Discussion

### Principal Findings of the Study

In this study, we found significant changes in the composition and metabolic function of vaginal microbiota in POI patients. The relative abundance of *Actinobacteria*, *Atopobium*, and *Gardnerella* were significantly increased in POI patients, while *Bifidobacterium* was significantly decreased. These alterations were significantly correlated with reduced ovarian reserve, endocrine disruption, and symptoms of perimenopausal syndrome in POI patients. The key bacterial taxa *Gardnerella* and *Atopobium* showed potency in predicting POI.

Different from the results of previous studies, we also found significant differences in the composition and metabolic function of vaginal microbiota in the POI (25 < FSH ≤ 40) and POF (FSH > 40) subgroups. *Firmicutes* was significantly reduced in POF (FSH > 40) patients, while *Actinobacteria* was significantly increased. And compared with the control women, *Firmicutes*, and *Lactobacillus* were significantly decreased only in POF (FSH > 40) patients, while no difference was observed in POI (25 < FSH ≤ 40) patients. Similarly, *Gardnerella* and *Atopobium* also showed potency in predicting POF (FSH > 40).

### Interpretation of the Findings

In recent years, a large number of studies have shown that the vaginal microbiota plays an important role in maintaining the health of the female reproductive system (32–34). Previous studies have indicated changes in the diversity and composition of the vaginal microbiota in postmenopausal women compared with premenopausal women, especially overall bacteria levels and the decrease in *Lactobacillus (* 22, 35, 36). Recently, two studies showed that the diversity and richness of the vaginal microbiota of POF patients were statistically different from healthy controls. Wang et al. found that the relative abundance of *Lactobacillus* was significantly reduced in POF patients and it is less abundant in postmenopausal women than in POF patients (23). Wu et al. also found a significant decrease in *Lactobacillus* in POI patients with FSH > 40 IU/L (24).

Accordingly, we also observed a decrease in *Lactobacillus* in POI patients, but the difference was not statistically significant. However, our subgroup analysis based on serum FSH levels found that the controls and POI (25 < FSH ≤ 40) patients were both had higher relative abundances of *Firmicutes* and *Lactobacillus* than POF (FSH > 40) patients. With an in-depth study of the etiology and the accumulation of clinical cases, doctors and researchers have gradually realized that ovarian aging is a group of diseases with diverse clinical manifestations, complex etiology, and progressive development, including the hidden stage, the biochemical abnormal stage, the clinical abnormal stage, and menopause. POF and POI represent different stages in the progression of ovarian aging (2, 37–39). POI is a disease state that occurs when ovarian function declines past a decisive point before the age of 40. To a certain extent, it reflects the diversity and progress of ovarian aging. However, menopause and POF can only represent the terminal stage of ovarian failure, they cannot reflect actual disease progression. In the definition of POI and POF, the diagnostic threshold of FSH is different. The diagnostic criterion of POF is FSH > 40 IU/L, while that of POI is FSH > 25 IU/L. The latter lowers the diagnostic threshold of FSH to identify POI patients as early as possible to achieve early diagnosis and early treatment. Our results suggest that the changes of vaginal microbiota, including *Lactobacillus*, may also progress gradually, similar to ovarian aging, but further longitudinal studies with larger sample size are needed to confirm this.

*Gardnerella* and *Atopobium*, which both belong to *Actinobacteria*, were found significantly enriched in POI patients in our study and were significantly correlated with declined ovarian reserve, endocrine disruption, and symptoms of perimenopausal syndrome. Similar increases have also been found in postmenopausal women and POF patients (23). However, Ouarabi et al. failed to find a significant difference in the abundance of *Gardnerella* and *Atopobium* between healthy childbearing and postmenopausal women (35). *Gardnerella* and *Atopobium* may be associated with ovarian function, but results from different ethnic populations are still controversial and need to be studied further. We also found that *Gardnerella* and *Atopobium* may have a certain potential in predicting POI and POF (FSH > 40). However, using vaginal microbiota alone as a predictor may not be a good differentiator between women with POI and bacterial vaginitis. Because bacterial vaginitis is also often characterized by the presence of *Gardnerella* and *Atopobium* in high abundance (40).

The relative abundance of *Bifidobacterium* was dramatically decreased in POI patients. *Bifidobacterium* mainly colonizes in the human intestines and is only presents at low levels in the vagina. As a type of probiotic, it has anti-inflammatory effects, improves immune function, and resists oxidative damage (41–43). Clinical studies have found that *Bifidobacterium* can improve the metabolism and cardiovascular function in postmenopausal women (44–46). Studies have also found that *Bifidobacterium* can regulate the secretion of sex hormones in PCOS patients and significantly reduce serum LH levels and LH/FSH ratio (47). There is currently no research taking place on probiotics in POI patients. Whether probiotics such as *Bifidobacterium* can delay the development of POI or contribute to the treatment of POI is still unknown and remains to be explored in the future. Moreover, owing to the influence of human internal factors and environmental factors, individual differences in the use of probiotics are relatively large. The mainstream trend in the future is personalized probiotic intervention strategies. This is also true for POI patients.

Our study also found that changes in vaginal microbiota were associated with symptoms and severity of the perimenopausal syndrome. This may be related to the decrease in *Lactobacillus* and the increase in anaerobic bacteria such as *Gardnerella* and *Atopobium.* Previous studies found that a decrease in *Lactobacillus* in the vagina after menopause was associated with vulvovaginal atrophy symptoms, such as vaginal interference, dyspareunia, and vaginal pain (22). Hummelen et al. found an inverse correlation between the relative abundance of *Lactobacillus* and vaginal dryness, and an increased bacterial diversity in women experiencing moderate to severe vaginal dryness (48). In addition, a study has also shown that HRT increased total bacterial and *Lactobacillus* in the vagina of postmenopausal women, and improved vagina atrophy symptoms (36). The relationship between these microbes and symptoms of perimenopausal syndrome needs further confirmation, and inventions regulating the vaginal microbiota may be a potential alternative therapy for ameliorating the symptoms.

### Strengths and limitations

Our study provided a comprehensive description of vaginal microbiota changes in POI patients, and reveal correlations between these changes and ovarian reserve, ovarian endocrine function, and perimenopausal symptoms. We also found differences in the vaginal microbiota between the POI (25 < FSH ≤ 40) and POF (FSH > 40) subgroups. Some limitations of the current study also merit careful consideration. The incidence of POI is relatively low, which limited our ability to perform a study with larger sample size and draw more conclusions. The practicality of recruiting much larger numbers would be difficult, especially for POI (25 < FSH ≤ 40) patients. Furthermore, since our study is a case-control design, the causal associations could not be inferred, and mechanistic insight was not provided. But our preliminary results provided a platform for further investigation of potential research queries. The identified bacteria and their metabolic function differences may provide insights into further research regarding the roles of the microbiome in the pathophysiology, diagnosis, and treatment of POI.

## Conclusions

In summary, our study revealed alterations in the vaginal microbiota in POI patients, and these changes were related to reduced ovarian reserve, endocrine disruption, and symptoms of perimenopausal syndrome. Differences in vaginal microbiota between POI (25 < FSH ≤ 40) and POF (FSH > 40) patients were also identified. The key bacterial taxa *Gardnerella* and *Atopobium* showed potency in predicting POI. Our findings added new evidence for the relationship between vaginal microbiota and ovarian function. In addition, subgroup analysis of the POI patients was conducted based on serum FSH levels, which can reflect the changes of vaginal microbiota in more detail during the ovarian function failure process. However, future studies are still needed to comprehensively verify the compositive changes of vaginal microbiota and explore the potential mechanisms underlying the associations between the vaginal microbiota and POI.

## Data Availability Statement

The data supporting the results of this article is available in the NCBI SRA repository. Accession to cite for these SRA data: PRJNA756767 (https://www.ncbi.nlm.nih.gov/sra/PRJNA756767).

## Ethics Statement

The studies involving human participants were reviewed and approved by the Ethical Committee of Tongji Medical College, Huazhong University of Science and Technology. The patients/participants provided their written informed consent to participate in this study.

## Author Contributions

JW, JZ, and SW conceived and designed the study. JW, YF, WY, SY, and AL recruited participants and collected their vaginal samples and related information. JW and JZ analyzed the data. JW and SW drafted and revised the paper. All authors contributed to the article and approved the submitted version of the manuscript.

## Funding

The study was supported by the grant from the National Natural Science Foundation of China (81873824, 81701438, and 82001498), and the Clinical Research Pilot Project of Tongji hospital, Huazhong University of Science and Technology (2019CR205).

## Conflict of Interest

The authors declare that the research was conducted in the absence of any commercial or financial relationships that could be construed as a potential conflict of interest.

## Publisher’s Note

All claims expressed in this article are solely those of the authors and do not necessarily represent those of their affiliated organizations, or those of the publisher, the editors and the reviewers. Any product that may be evaluated in this article, or claim that may be made by its manufacturer, is not guaranteed or endorsed by the publisher.

## Supplementary Material

Supplementary Figure 1 Comparison of vaginal microbiota diversity in women with POI and healthy controls. **(A–E)** Comparison of alpha diversity index Observed-OTUs, Chao 1 index, Shannon index, Simpson index, and Goods_coverage. **(F)** Beta diversity was significantly different between the two groups according to Anosim (*R* = 0.0634, *P* = 0.027) and is shown in PCA plots. OTU, operational taxonomic unit; Anosim, analysis of similarities; PCA, principal component analysis.

Supplementary Figure 2 Composition of the vaginal microbiota of POI patients and controls. Relative abundance is shown at the phylum **(A)**, class **(B)**, order **(C)**, family **(D)**, genus **(E)**, and species **(F)** levels. **(G–I)**. Differences in relative abundance at class, order, and family levels. *P* < 0.05 was considered statistically significant.

Supplementary Figure 3 The correlation coefficient between the top 20 most abundant genera in all samples.

Supplementary Figure 4 Analyze and predict the metabolic functions of vaginal microbiota by the PICRUSt2 method. **(A)** Top 20 significantly different pathways between the control group and the POI (25 < FSH ≤ 40) subgroup. **(B)** Top 20 significantly different pathways between the control group and the POF (FSH > 40) subgroup. *P* < 0.05 was considered statistically significant.

Supplementary Figure 5 No Spearman’s correlation was found between alpha diversity indices of vaginal microbiota and ovarian reserve, ovarian function, and KMI score. PRL, prolactin; AMH, anti-müllerian hormone; E2, estradiol; LH, luteinizing hormone; FSH, follicle-stimulating hormone; KMI, Kupperman index; PRG, progesterone; T, testosterone.

Supplementary Figure 6 The potential of key bacterial taxa to predict POF (FSH > 40). ROC curves were constructed and AUC values were calculated to assess the potential of predicting POF (FSH > 40) for *Actinobacteria* **(A)**, *Gardnerella* **(B)**, and *Atopobium* **(C)**. ROC, receiver operating characteristic; AUC, area under the curve.

Supplementary Figure 7 The potential of *Atopobium* to predict POI (25 < FSH ≤ 40). AUC, area under the curve.

Supplementary Table 1 Basic clinical characteristics of the participants.

Supplementary Table 2 Phylum abundance: all.

Supplementary Table 3 Genus abundance: top 20.

Supplementary Table 4 Species abundance: top 20.

Supplementary Table 5 Spearman correlation analyses of passive smoking, age at menarche, and vaginal microbiota.
