# Supplementary figures and images for: Vaginal Microbiota Changes in Patients With Premature Ovarian Insufficiency and Its Correlation With Ovarian Function
Source: Front Endocrinol (Lausanne). 2022 Feb 22;13:824282. doi: 10.3389/fendo.2022.824282 (PMC8902819; doi:10.3389/fendo.2022.824282)

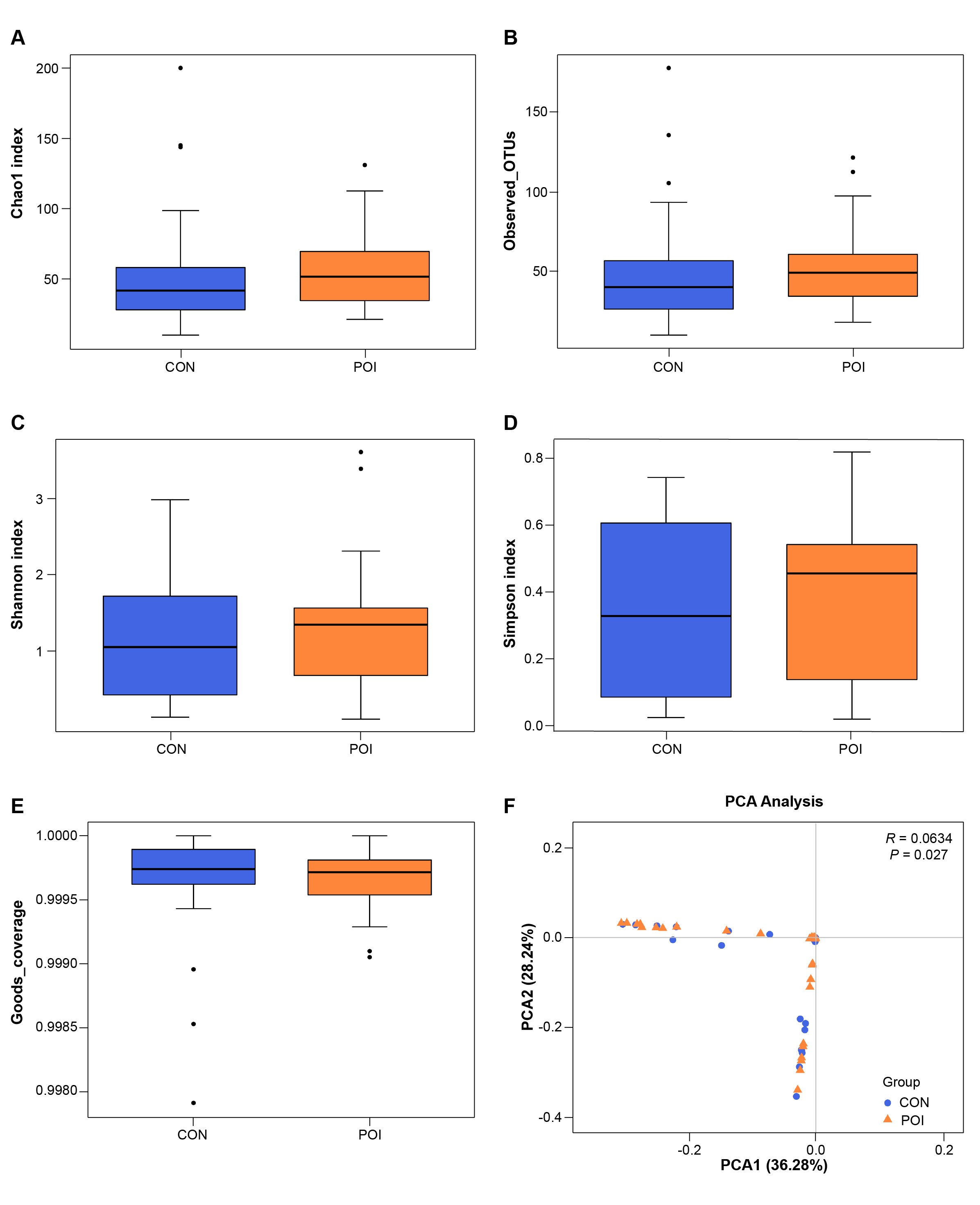

Supplement: Supplementary Figure 1 — Comparison of vaginal microbiota diversity in women with POI and healthy controls. (A–E) Comparison of alpha diversity index Observed-OTUs, Chao 1 index, Shannon index, Simpson index, and Goods_coverage. (F) Beta diversity was significantly different between the two groups according to Anosim (R = 0.0634, P = 0.027) and is shown in PCA plots. OTU, operational taxonomic unit; Anosim, analysis of similarities; PCA, principal component analysis. [file Image_1.tif]

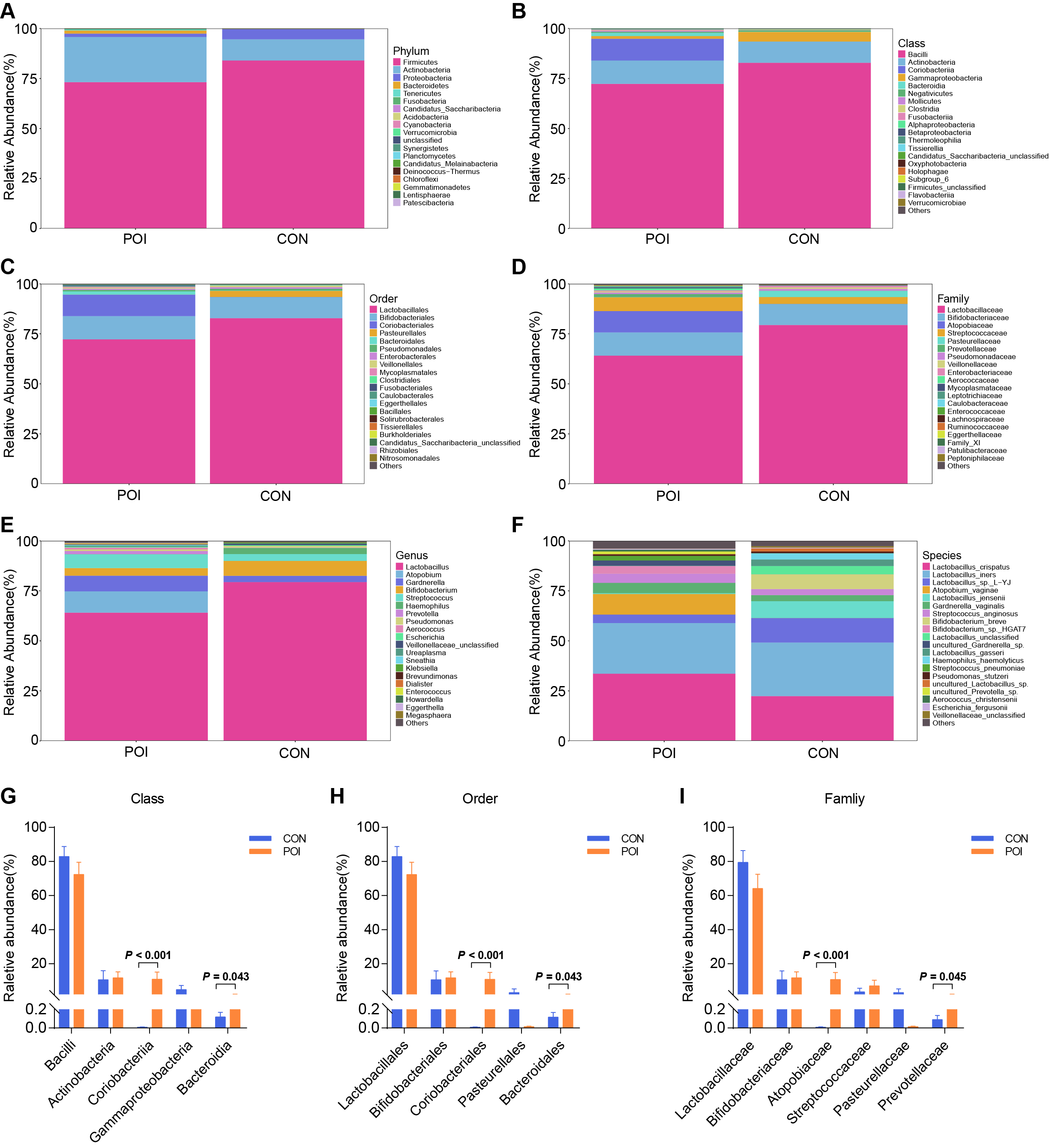

Supplement: Supplementary Figure 2 — Composition of the vaginal microbiota of POI patients and controls. Relative abundance is shown at the phylum (A), class (B), order (C), family (D), genus (E), and species (F) levels. (G–I). Differences in relative abundance at class, order, and family levels. P < 0.05 was considered statistically significant. [file Image_2.tif]

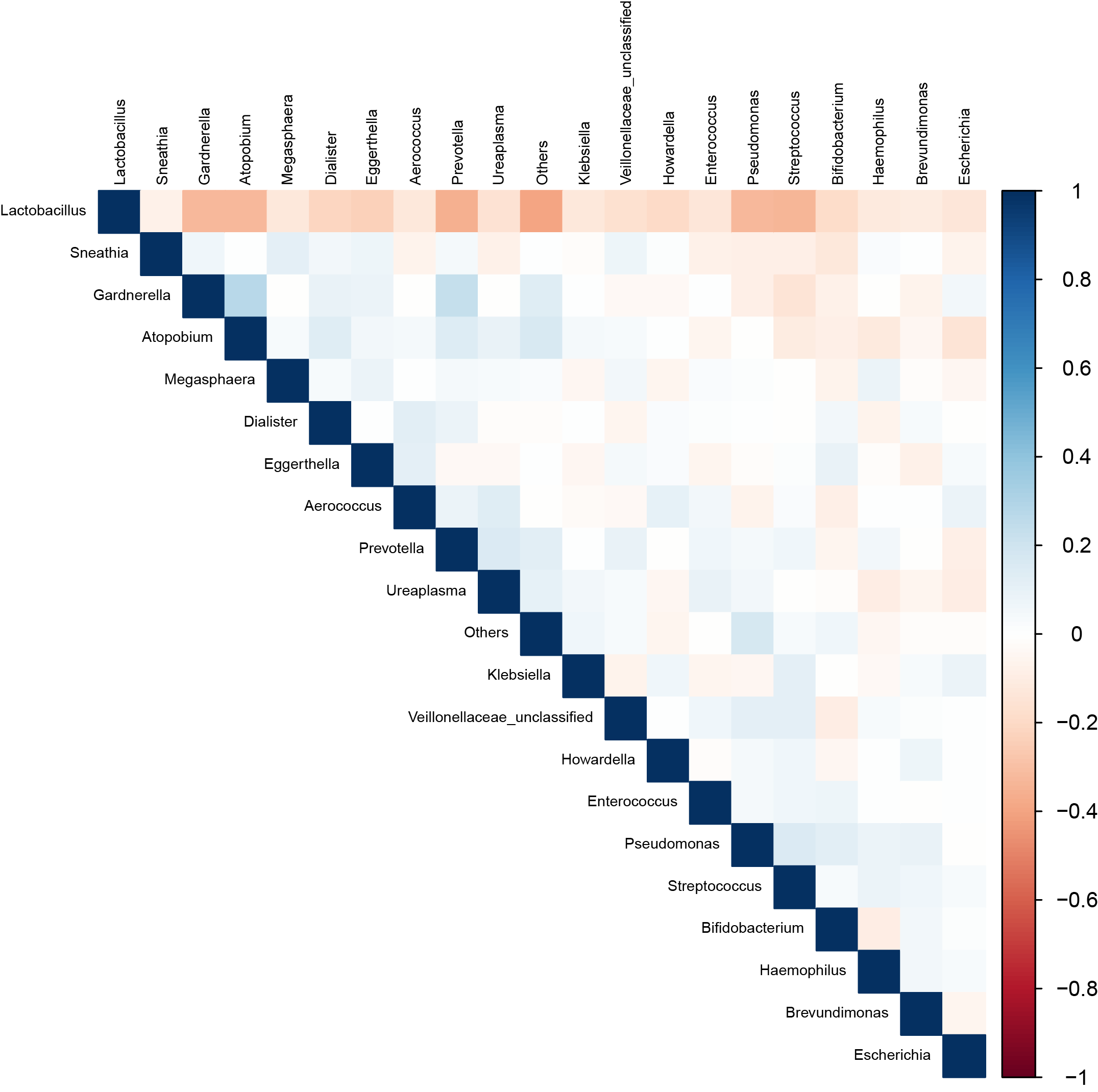

Supplement: Supplementary Figure 3 — The correlation coefficient between the top 20 most abundant genera in all samples. [file Image_3.tif]

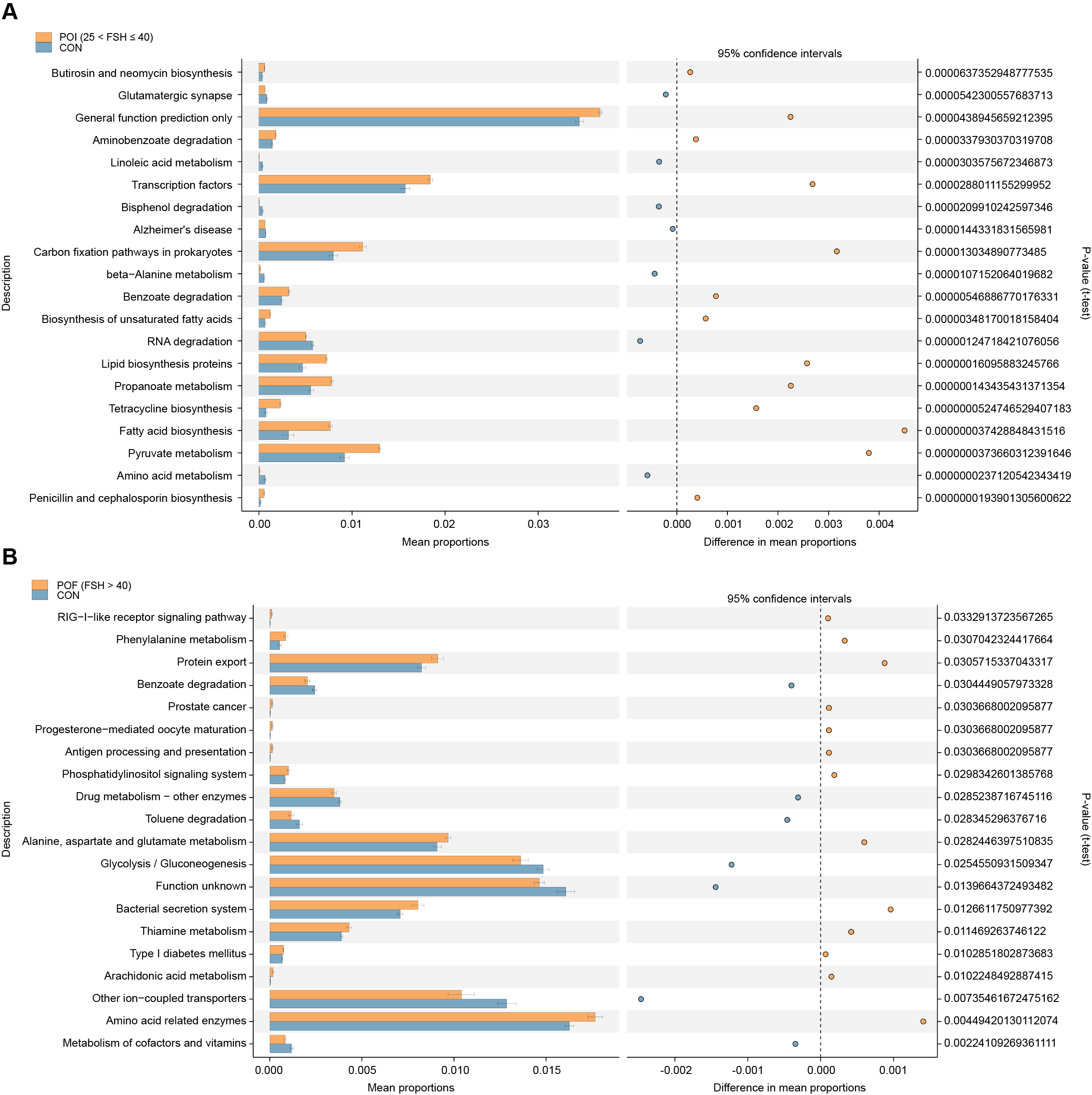

Supplement: Supplementary Figure 4 — Analyze and predict the metabolic functions of vaginal microbiota by the PICRUSt2 method. (A) Top 20 significantly different pathways between the control group and the POI (25 < FSH ≤ 40) subgroup. (B) Top 20 significantly different pathways between the control group and the POF (FSH > 40) subgroup. P < 0.05 was considered statistically significant. [file Image_4.tif]

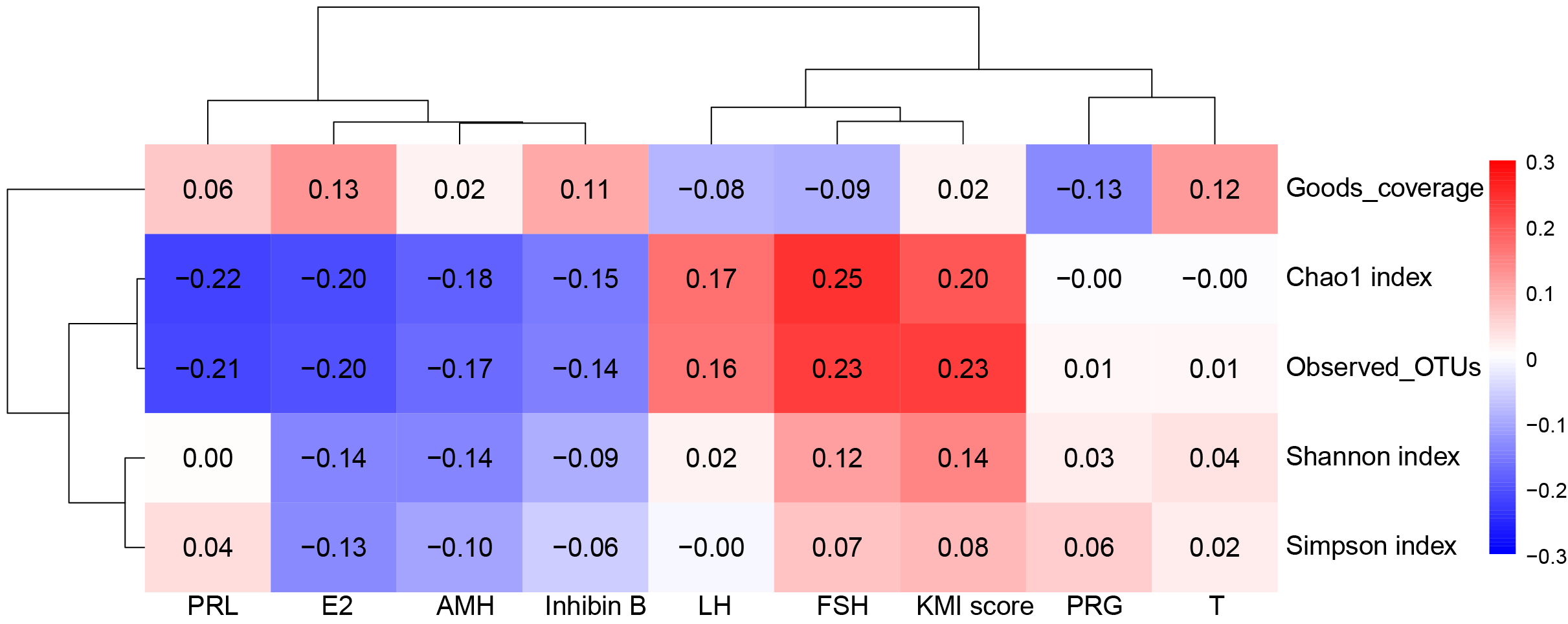

Supplement: Supplementary Figure 5 — No Spearman’s correlation was found between alpha diversity indices of vaginal microbiota and ovarian reserve, ovarian function, and KMI score. PRL, prolactin; AMH, anti-müllerian hormone; E2, estradiol; LH, luteinizing hormone; FSH, follicle-stimulating hormone; KMI, Kupperman index; PRG, progesterone; T, testosterone. [file Image_5.tif]

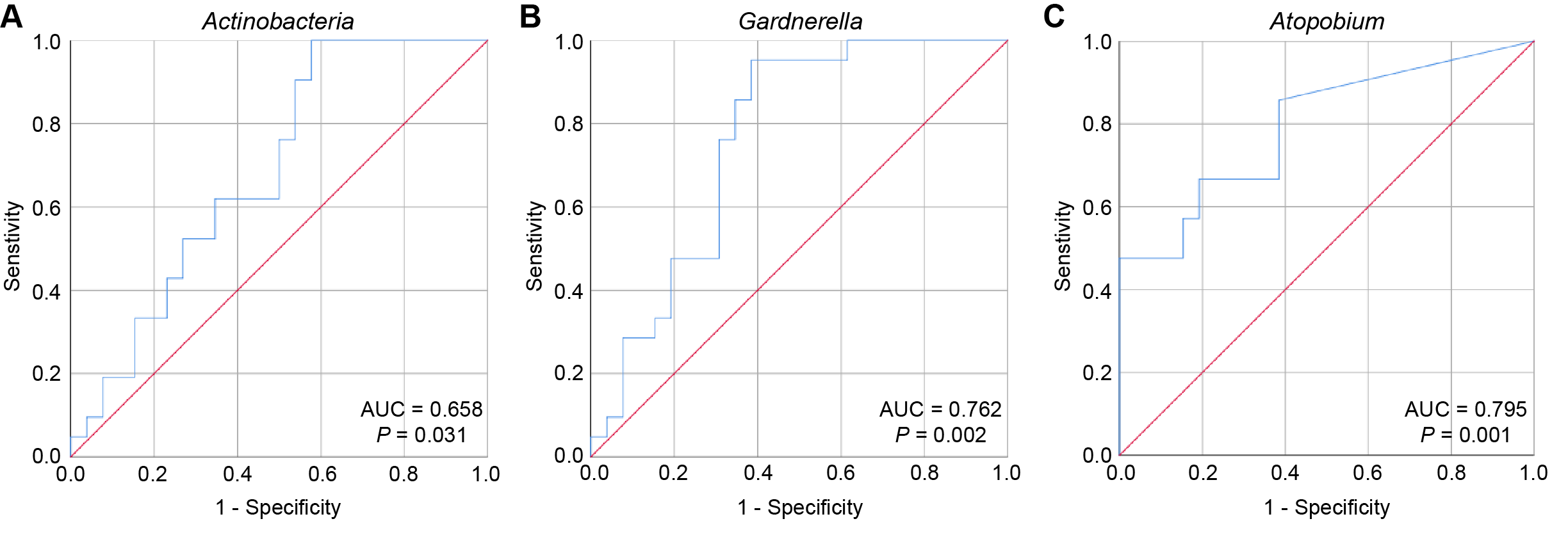

Supplement: Supplementary Figure 6 — The potential of key bacterial taxa to predict POF (FSH > 40). ROC curves were constructed and AUC values were calculated to assess the potential of predicting POF (FSH > 40) for Actinobacteria (A), Gardnerella (B), and Atopobium (C). ROC, receiver operating characteristic; AUC, area under the curve. [file Image_6.tif]

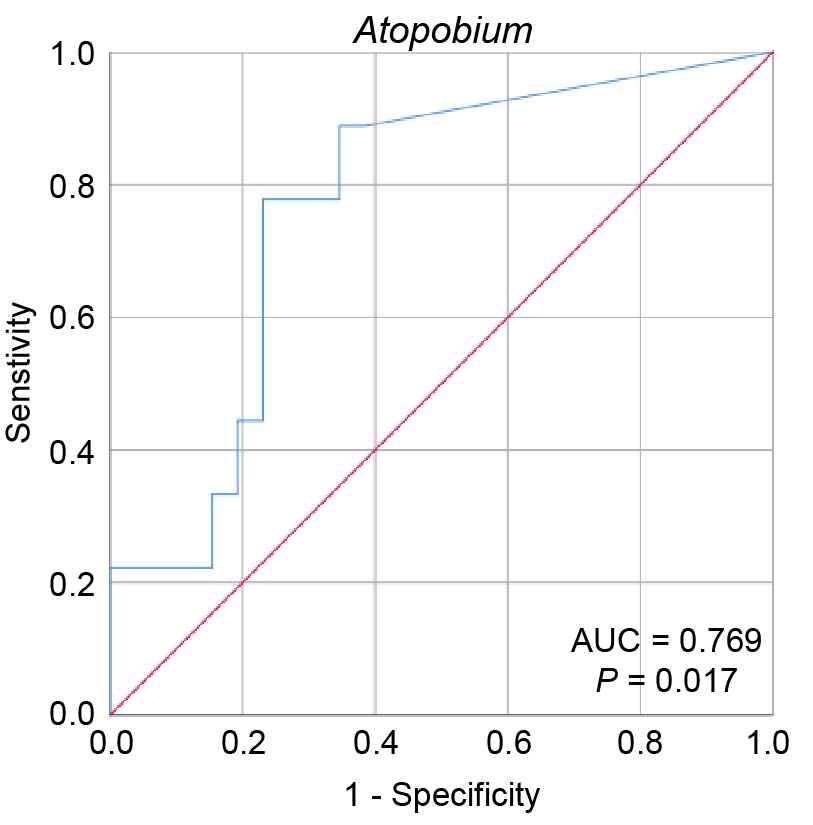

Supplement: Supplementary Figure 7 — The potential of Atopobium to predict POI (25 < FSH ≤ 40). AUC, area under the curve. [file Image_7.tif]
