# Supplementary material for: Vaginal Microbiota Changes in Patients With Premature Ovarian Insufficiency and Its Correlation With Ovarian Function
Source: Front Endocrinol (Lausanne). 2022 Feb 22;13:824282. doi: 10.3389/fendo.2022.824282 (PMC8902819; doi:10.3389/fendo.2022.824282)
Supplement: Supplementary Table 1 — Basic clinical characteristics of the participants. [file Table_1.doc]

Table S1. Basic clinical characteristics of the participants.

| Group | CON (n=26) | POI (n=30) | *P* value |
| --- | --- | --- | --- |
| **Demographics** |  |  |  |
| Age, median (IQR), years | 33 (28.75-38.25) | 35.5 (29-38.25) | 0.810 |
| BMI, mean±SD, kg/m^2^ | 20.89±2.39 | 21.49±2.97 | 0.414 |
| Nationality, n (%) |  |  |  |
| Han | 26(100) | 28(93.3) | 0.494 |
| Minority | 0(0) | 2(6.7) |  |
| Residence, n (%) |  |  |  |
| Urban | 22(84.6) | 25(83.3) | 1.000 |
| Rural | 4(15.4) | 5(16.7) |  |
| Advanced education, n (%) |  |  |  |
| Yes | 12(46.2) | 16(53.3) | 0.592 |
| No | 14(53.8) | 14(46.7) |  |
| Employed, n (%) |  |  |  |
| Yes | 23(88.5) | 25(83.3) | 0.712 |
| No | 3(11.5) | 5(16.7) |  |
| Monthly income, n (%) |  |  |  |
| ≤10000 | 17(65.4) | 25(83.3) | 0.122 |
| ＞10000 | 9(34.6) | 5(16.7) |  |
| **Lifestyles** |  |  |  |
| Smoking, n (%) |  |  |  |
| Yes | 0(0) | 3(10) | 0.240 |
| No | 26(100) | 27(90) |  |
| Passive smoking, n (%) |  |  |  |
| Yes | 7(26.9) | 16(53.3) | **0.045** |
| No | 19(73.1) | 14(46.7) |  |
| Alcohol drinking, n (%) |  |  |  |
| Yes | 12(46.2) | 8(26.7) | 0.129 |
| No | 14(53.8) | 22(73.3) |  |
| Tea drinking, n (%) |  |  |  |
| Yes | 5(19.2) | 6(20) | 0.942 |
| No | 21(80.8) | 24(80) |  |
| Exercise, n (%) |  |  |  |
| Yes | 11(42.3) | 12(40) | 0.861 |
| No | 15(57.7) | 18(60) |  |
| Regular work and rest, n (%) |  |  |  |
| Yes | 17(65.4) | 13(43.3) | 0.099 |
| No | 9(34.6) | 17(56.7) |  |
| Sleep duration per day, n (%) |  |  |  |
| 6 hours | 6(23.1) | 8(26.7) | 0.757 |
| ≥7 hours | 20(76.9) | 22(73.3) |  |
| Toxic substance exposure, n (%) |  |  |  |
| Yes | 3(11.5) | 4(13.3) | 1.000 |
| No | 23(88.5) | 26(86.7) |  |
| **Reproductive histories** |  |  |  |
| Age at menarche, median (IQR), years | 13 (12.75-15) | 12 (12-13.25) | **0.016** |
| Gravidity, median (range) | 1.5 (0-7) | 2 (0-7) | 0.657 |
| Category, n (%) |  |  |  |
| 0 | 8(30.8) | 9(30) | 0.331 |
| 1-2 | 6(23.1) | 12(40) |  |
| ≥3 | 12(46.2) | 9(30) |  |
| Induced abortion, median (range) | 0 (0-6) | 1 (0-7) | 0.545 |
| Category, n (%) |  |  |  |
| 0 | 16(61.5) | 14(46.7) | 0.266 |
| ≥1 | 10(38.5) | 16(53.3) |  |
| Age at first pregnancy, mean±SD, years | 23.00±3.48 | 22.52±2.29 | 0.612 |
| Age at first birth, mean±SD, years | 24.81±3.54 | 24.17±2.62 | 0.547 |
| **Sex hormones** |  |  |  |
| FSH, mean±SD, (mIU/ml) | 5.98±1.11 | 78.08±44.79 | **＜0.001** |
| LH, median (IQR), (mIU/ml) | 3.64 (3.11-5.87) | 36.99 (19.07-52.65) | **＜0.001** |
| PRL, median (IQR), (ng/ml) | 10.91 (9.32-13.64) | 9.54 (6.61-15.28) | 0.362 |
| PRG, median (IQR), (ng/ml) | 0.54 (0.43-0.79) | 0.44 (0.30-0.77) | 0.063 |
| T, median (IQR), (ng/ml) | 37.96 (27.16-45.19) | 18.49 (0.62-32.50) | **＜0.001** |
| E2, median (IQR), (pg/ml) | 40.18 (36.61-63.54) | 32.98 (19.44-38.45) | **＜0.001** |
| AMH, median (IQR), (ng/ml) | 3.99 (2.51-5.15) | 0.12 (0.06-0.18) | **＜0.001** |
| Inhibin B, median (IQR), (pg/ml) | 111.63 (97.38-133.51) | 12.46 (10.00-17.94) | **＜0.001** |
| **KMI score**, Mean±SD | 7.77±3.96 | 13.90±8.62 | **0.001** |
| Category, n (%) ^a^ |  |  |  |
| ≤6 | 10 (38.5) | 8 (26.7) | **0.003** |
| 7-15 | 16 (61.5) | 11 (36.7) |  |
| ＞16 | 0(0) | 11(36.7) |  |
| **HAD score** |  |  |  |
| Anxiety score, mean±SD | 3.04±2.69 | 4.17±4.93 | 0.285 |
| Category, n (%) ^b^ |  |  |  |
| 0-7 | 25 (96.2) | 23(76.7) | 0.056 |
| ＞7 | 1(3.8) | 7 (23.3) |  |
| Depression score, mean±SD | 3.58±3.02 | 4.97±3.92 | 0.148 |
| Category, n (%) ^b^ |  |  |  |
| 0-7 | 24 (92.3) | 23 (76.7) | 0.154 |
| ＞7 | 2 (7.7) | 7 (23.3) |  |

^a^ KMI scores ranging from 0-6, 7-15, and﹥16 were used to rate the degree of severity as none, mild and moderate to severe, respectively.

^b^ HAD scores ranging from 0-7, and﹥7 represented normal and anxiety/depression, respectively.

IQR, interquartile; SD, standard deviation; BMI, body mass index; FSH, follicle stimulating hormone; LH, luteinizing hormone; PRL, prolactin; PRG, progesterone; T, testosterone; E2, estradiol; AMH, anti-Müllerian hormone; KMI, Kupperman index; HAD, hospital anxiety and depression.
