# Supplementary material for: Vaginal Microbiota Changes in Patients With Premature Ovarian Insufficiency and Its Correlation With Ovarian Function
Source: Front Endocrinol (Lausanne). 2022 Feb 22;13:824282. doi: 10.3389/fendo.2022.824282 (PMC8902819; doi:10.3389/fendo.2022.824282)
Supplement: Supplementary Table 5 — Spearman correlation analyses of passive smoking, age at menarche, and vaginal microbiota. [file Table_5.docx]

Table S5. Spearman correlation analyses of passive smoking, age at menarche, and vaginal microbiota.

|  |  | Passive smoking | | Age at menarche | |
| --- | --- | --- | --- | --- | --- |
|  |  | *r* | *P*-value | *r* | *P*-value |
| **Alpha diversity** | Observed_OTUs | **-0.34** | **0.010** | 0.06 | 0.651 |
|  | Chao1 index | **-0.35** | **0.009** | 0.05 | 0.741 |
|  | Shannon index | -0.15 | 0.272 | 0.05 | 0.699 |
|  | Simpson index | -0.11 | 0.424 | 0.02 | 0.885 |
|  | Goods_coverage | 0.14 | 0.316 | 0.03 | 0.849 |
| **Phylum** | *Firmicutes* | 0.21 | 0.129 | -0.05 | 0.722 |
|  | *Actinobacteria* | -0.12 | 0.387 | 0.07 | 0.612 |
|  | *Proteobacteria* | 0.02 | 0.902 | -0.04 | 0.774 |
| **Genus** | *Lactobacillus* | 0.26 | 0.056 | -0.06 | 0.684 |
|  | *Bifidobacterium* | **0.28** | **0.038** | -0.11 | 0.429 |
|  | *Gardnerella* | -0.09 | 0.493 | 0.05 | 0.690 |
|  | *Atopobium* | -0.07 | 0.602 | -0.03 | 0.829 |
|  | *Streptococcus* | -0.22 | 0.097 | 0.02 | 0.880 |

OTU, operational taxonomic unit.
